# Risk Factors for Recurrence of Symptomatic Common Bile Duct Stones after Cholecystectomy

**DOI:** 10.1155/2012/417821

**Published:** 2012-09-06

**Authors:** Ju Hyun Oak, Chang Nyol Paik, Woo Chul Chung, Kang-Moon Lee, Jin Mo Yang

**Affiliations:** Department of Internal Medicine, St. Vincent's Hospital, College of Medicine, The Catholic University of Korea, Ji-dong, Paldal-gu, Gyeonggi-do, Suwon 442-723, Republic of Korea

## Abstract

*Purpose*. The recurrence of CBD stone is still observed in a considerable number of patients. The study was to evaluate the risk factors for recurrence of symptomatic CBD stone in patients who underwent cholecystectomy after the removal of CBD stone. *Methods*. The medical records of patients who underwent removal of CBD stone with subsequent cholecystectomy were reviewed. The risk factors for the recurrence of symptomatic CBD stone were compared between the recurrence and the nonrecurrence group. *Results*. The mean follow-up period was 40.6 months. The recurrence of symptomatic CBD stones was defined as the detection of bile duct stones no sooner than 6 months after complete clearance of CBD stones, based on symptoms or signs of biliary complication. 144 patients (68 males, 47.2%) were finally enrolled and their mean age was 59.8 (range: 26*~*86) years. The recurrence of CBD stone occurred in 15 patients (10.4%). The mean period until first recurrence was 25.9 months. The presence of type 1 or 2 periampullary diverticulum and multiple CBD stones were the independent risk factors. *Conclusion*. For the patients with type 1 or 2 periampullary diverticulum or multiple CBD stones, careful followup is needed for the risk in recurrence of symptomatic CBD stone.

## 1. Background

After a removal of common bile duct (CBD) stone, cholecystectomy is performed to prevent biliary colic, cholecystitis, pancreatitis, or CBD stone recurrence [1–3]. CBD stones usually originate in the gallbladder and then they migrate [4]. Therefore subsequent cholecystectomy would be helpful to prevent CBD stone recurrence. However recurrence of CBD stone is still observed in a considerable number of patients following cholecystectomy. Bile duct stones that are demonstrated 6 months or more after endoscopic retrograde cholangiopancreatography (ERCP) are considered to be recurrent [3, 5, 6]. There have been several studies about the prevalence of and the risk factors for CBD stone recurrence after endoscopic sphincterectomy [7–12], but little data is available on CBD stone recurrence after cholecystectomy. The risk factors for recurrent bile duct stones were known as a dilated common bile duct, gall bladder stone, biliary stricture, angulation of the CBD, previous open cholecystectomy, and periampullary diverticulum [7, 8, 10, 12, 13]. Considering the periampullary diverticulum in detail, the recent study [8] suggested that specific status in which papilla located within or on the inner rim of the diverticulum was associated with recurrence. This study aimed to evaluate the risk factors for CBD stone recurrence in patients who underwent cholecystectomy after the removal of the initial CBD stones. 

## 2. Materials and Methods

### 2.1. Patients

The study was conducted at St. Vincent's Hospital, a teaching hospital of the Catholic University School of Medicine. We surveyed the patients who underwent endoscopic or surgical removal of CBD stone and then subsequent cholecystectomy within a month at our hospital between January 2005 and February 2010. The inclusion criteria were an age older than 18 years. Exclusion criteria were no history of prior CBD stone, hemolytic anemia, inflammatory bowel disease (Crohn's disease, ulcerative colitis, etc.) severe liver diseases (liver cirrhosis, hepatocellular carcinoma), biliary malignancy such as gall bladder cancer or cholangiocarcinoma, abdominal surgery for the liver or the pancreatobiliary system, and no evidence of stenosis of the bile duct. Complete removal of CBD stone was confirmed either by follow-up ERCP or an intraoperative cholangiogram during the cholecystectomy. The recurrence of CBD stones was defined as the development of stones according to ERCP not earlier than 6 months after the confirmation of complete removal of the CBD stones. The exclusion criteria specified a recurrence of CBD stone within 6 months after cholecystectomy.

### 2.2. ERCP and Operation

For the all patients considered to have symptomatic CBD stones, endoscopic sphincterotomy during the ERCP was performed with standard techniques and using a sphincterotome or a needle knife, and the stones were extracted with baskets or retrieval balloons, and lithotripsy was used for large stones. The complete CBD stone extraction was confirmed via endoscopic cholangiography by the endoscopist and radiologist, and this was followed by performing subsequent cholecystectomy. If endoscopic stone removal was impossible because of the stone's size, impacted stone, the presence of periampullary diverticulum, and/or the reluctance to undergo a maintaining procedure due to poor compliance or severe illness, then laparoscopic or open surgical stone removal with cholecystectomy was performed. Complete stone extraction was confirmed via a choledochoscope or an intraoperative cholangiogram. 

### 2.3. Study Process

We retrospectively surveyed the patients' chart and the digitalized picture archiving communication system (PACS). The patients were divided into 2 groups. The one group had recurrence of symptomatic CBD stone (the recurrence group), and the other group was free of signs or symptoms of stone recurrence (the nonrecurrence group), after the removal of stone with subsequent cholecystectomy, during the follow-up period. The decision on the need for subsequent ERCP for detecting the recurrence of CBD stone during the follow-up period was made by a gastroenterologist based on the recurrence of signs or symptoms of biliary complications. These indications included (1) obstructive jaundice, which was defined as elevation of the serum liver enzymes and bilirubin with an etiology assumed to be CBD stones, sludge or CBD dilation by radiologic study such as abdomen ultrasonography (US), computed tomography (CT), or magnetic resonance cholangiopancreatography (MRCP) (2) acute biliary pancreatitis, and (3) acute cholangitis. The follow up period was from the date of the initial complete clearance of CBD stones to the date of the visit to the hospital for recurrence of CBD stones or February, 2010 for the nonrecurrence group. We interviewed the nonrecurrence group by telephone to identify the symptoms recurred if the patients' follow-up data was missed. 

The primary outcomes were the incidence and risk factors of CBD stone recurrence. These were evaluated after cholecystectomy following complete clearance of CBD stone. The following variables were recorded for all the patients; age, gender, comorbid disease (diabetes or hypertension), smoking, the alcohol history, and the laboratory data. The CBD stone size (the largest in the case with multiple stones), the number of stones, the CBD diameter at its widest point, the distal CBD angle, the distal CBD length, the presence of juxtaampullary diverticulum, the presence of intrahepatic duct (IHD) stone, and a history of undergoing lithotripsy were reviewed according to the initial ERCP findings. The types of operation (laparoscopic or open cholecystectomy) were also surveyed. Periampullary diverticulum was defined as the presence of a diverticulum within a 2 cm radius from the major papilla. It was divided into 3 types as type 1: the papilla was located within the diverticulum, type 2: the papilla was located on the inner rim of the diverticulum, and type 3: the papilla was located outside of the diverticulum [14]. The CBD diameter and the distal CBD angle and length were measured on the cholangiogram immediately after stone removal, with the patients in the prone position. The secondary outcomes were the probability curve for the patients remaining free of the recurrence of symptomatic CBD stone after the complete removal of the initial CBD stones with subsequent cholecystectomy according to the significant risk factors.

### 2.4. Statistical Analysis

The data is expressed as means ± standard deviations. The categorical variables were analyzed using independent sample *t*-tests or chi-square tests. Stepwise logistic regression analysis was used to identify the independent risk factors for the recurrence of symptomatic CBD stone. The odds ratios and 95% confidence intervals were calculated. Using the significant risk factors confirmed by multivariate analysis, the actuarial probability curves for patients remaining free of recurrence of symptomatic CBD stone were constructed using the Kaplan-Meier method, and these curves were compared with the log-rank test. The analyses were performed using a statistical software package (SPSS, version 13.0; SPSS Inc., Chicago, IL, USA). All *P* values less than 0.05 were considered significant for all tests. 

### 2.5. Ethical Considerations

This research adhered to the Declaration of Helsinki. The protocol of this study was approved by the Institutional Review Board of the Catholic University of Korea (VC11RISI0098). 

## 3. Results

### 3.1. Patients

During the study period, the removal of CBD stone with subsequent cholecystectomy was performed in 207 consecutive patients. Of these 207 patients, 63 were excluded due to the occurrence of CBD stone within 6 months after the complete removal of CBD stone (*n* = 12), underlying malignancy (cholangiocarcinoma, *n* = 1), congenital anomaly (choledochal cyst, *n* = 1), incomplete medical records (*n* = 2), failure of ERCP to confirm the CBD stone due to a poor general status (*n* = 4), follow-up loss, and unavailable to contact by telephone (*n* = 43). A total of 144 patients were finally enrolled in this study. The mean age of the patients (*n* = 144) was 59.8 (range: 26–86) years, and 68 (47.2%) were men. Mean follow-up period was 40.6 months. The recurrence of CBD stone occurred in 15 of 144 patients (10.4%) during the follow-up period, and the mean period until the first recurrence was 25.9 months. The characteristics of the patients in the two groups are shown in Table 1. The mean age is significantly higher in the recurrence group than that in the nonrecurrence group.

### 3.2. Risk Factors for the Recurrence of Symptomatic CBD Stone

On the univariate analysis, old age (*P* = 0.05), 2 or more CBD stones (*P* = 0.01), the presence of type 1 or type 2 diverticula (*P* = 0.03) and IHD stone (*P* = 0.05) were found to be significant related to recurrence of CBD stone (Table 2). A CBD stone size 10 mm or larger (*P* = 0.1) had a tendency to be correlated with stone recurrence. On the multivariate logistic regression analysis, multiple CBD stones ≥2 (*P* = 0.04) and the presence of type 1 or 2 periampullary diverticulum (*P* = 0.02) were the independent risk factors. The presence of IHD stone (*P* = 0.09) had a higher tendency for CBD stone recurrence (Table 3).

### 3.3. Probability of Patients Remaining Free of Recurrence of Symptomatic CBD Stone

**Figure 1:**
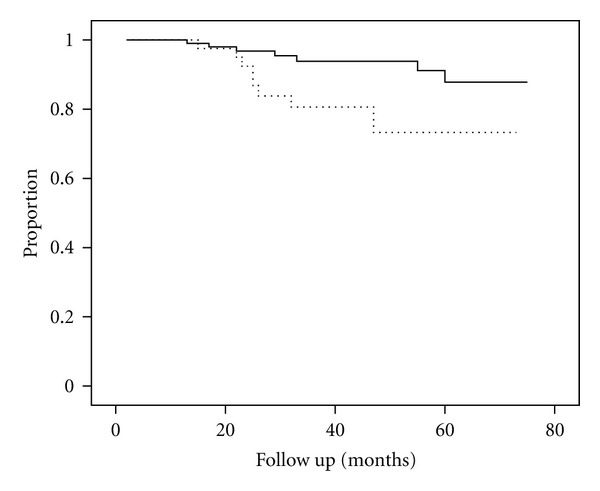
Actuarial probability curve of remaining free of recurrent symptomatic CBD stone after complete removal of the initial CBD stone with subsequent cholecystectomy. The patients with type 1 or 2 diverticulum versus those without diverticulum or those with type 3 diverticulum (80.5% versus 93.2%, resp.; *P* = 0.02, log-rank test) (- - -The patients with Type 1 or 2 diverticulum, or —the patients with type 3 diverticulum or without diverticulum).

**Figure 2:**
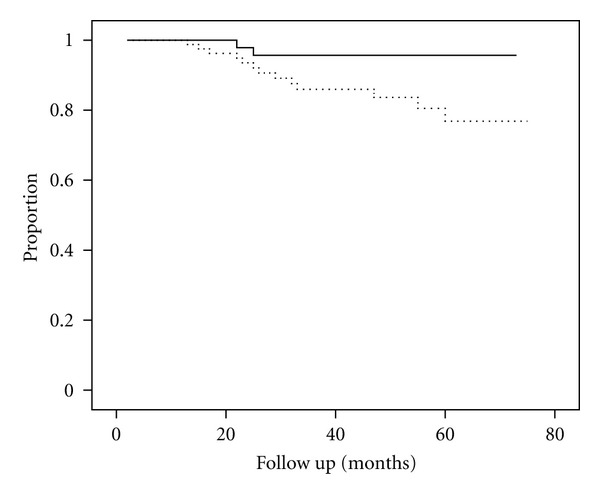
Actuarial probability curve of remaining free of recurrent symptomatic CBD stone after complete removal of the initial CBD stone with subsequent cholecystectomy. The patients with single CBD stone versus those with multiple CBD stones (96.8% versus 84.0%, resp.; *P* = 0.04, log-rank test) (- - -The patients with multiple CBD stones, or —the patients with single CBD stone).

The actuarial probability of patients remaining free of recurrence of symptomatic CBD stone during the followup after complete removal of the initial CBD stone with subsequent cholecystectomy for the patients with a single CBD stone was significantly higher than that for the patients with multiple CBD stones (≥2) (96.8% versus 84.0%, respectively; *P* = 0.04, log-rank test) (Figure 1). The patients with type 1 or 2 diverticulum had significantly lower rates of being free of recurrence of CBD stone during the followup than did the patients without diverticulum or who had only the type 3 diverticulum (80.5% versus 93.2%, resp.; *P* = 0.02, log-rank test) (Figure 2). 

## 4. Discussion

 The purpose of this study was to evaluate the prevalence and risk factors for recurrence of symptomatic CBD stones in patients with complete removal of the initial CBD stone and this was followed by subsequent cholecystectomy. The rate of recurrence of symptomatic CBD stone was 10.4% (15/144), and the significant risk factors were the presence of multiple CBD stones and type 1 or 2 periampullary diverticulum. 

The recurrence rate of CBD stone is reported to range from 4% to 24% [3, 5, 7–9, 11] which is consistent with our data. However most of the previous studies focused on the secondary CBD stones with the GB in situ, and studies on patients with recurrence of CBD stone who have undergone subsequent cholecystectomy are rare. Bile duct stones can recur after cholecystectomy because stones are formed in the bile duct in situ. This study included only patients who underwent complete clearance of initial CBD stones with subsequent cholecystectomy. 

There are no definite guidelines for following up patient with removed CBD stone who underwent subsequent cholecystectomy. In clinical practice, a considerate number of patients visit the hospital for management of the recurrence of symptomatic CBD stone. In this situation, identifying the risk factors for the development of recurrent CBD stones is needed. In this study, the independent risk factors for the recurrence of symptomatic CBD were type 1 or 2 periampullary diverticulum, and multiple CBD stones. 

The risk factors for recurrent bile duct stones after EST were previously suggested to be a dilated common bile duct, GB stone, periampullary diverticulum, biliary stricture, angulation of the CBD, previous open cholecystectomy, and lithotripsy [7, 8, 10, 12, 13]. However, in patients who underwent subsequent cholecystectomy, few data with small numbers of enrolled patients suggested that the risk factors for recurrent stones were the dilated CBD and periampullary diverticulum [8]. One report [13] suggested that the risk factors for recurrent CBD stones were more common in the elderly patients. In our study, old age was related to recurrence on the univariate analysis, but it was not associated with recurrence on the multivariate analysis. On the multivariate analysis, the independent factors for the recurrence of symptomatic CBD stone in patients with complete clearance of their initial CBD stone with subsequent cholecystectomy were the presence of multiple CBD stones and type 1 or 2 periampullary diverticulum at the time of the initial ERCP. The presence of IHD stone (*P* = 0.09) was not significant, but they had a tendency to be correlated with CBD stone recurrence. Patients with periampullary diverticulum have slow biliary emptying and bile stasis, which are important factors in bile duct stones formation [15]. Periampullary diverticulum has been advocated as a factor for recurrence of CBD stones in several previous studies [15–17], yet this is still controversial. One report [17] suggested that periampullary diverticulum is associated for patients with primary common bile duct stones, but not with the secondary ones. In our study, the presence of diverticulum alone was not related to recurrence, but the specific types such as type 1 or 2, with the papilla located on the inner rim of or within the diverticulum, were correlated with recurrence. The literature [8] suggested that type 1 as well as type 2 were related with recurrence, which is consistent with the results of our study. The factor of multiple CBD stones was considered as an independent risk factor for recurrence in our study. The anatomical factors of the CBD such as the extent of the diameter, angle, and length were not different between the recurrence and nonrecurrence groups. To exclude the retained stones that could be a factor for the development of symptomatic CBD stones, we analyzed the last imaging of cholangiography together with the radiologist after removal of the CBD stones. The unexplained unfavorable conditions to form stones such as the status of bacteria or the composition of bile need to be investigated. 

Two types of secondary CBD stones can be expected from the gall bladder and from IHD stone [18]. Our study included the patients with IHD stone because the chance to face IHD stone is not uncommon in clinical practice. A fair number of patients with concomitant IHD stones do not undergo a procedure to eliminate the IHD stone due to initial complete relief of biliary symptoms after the extraction of CBD stones, the difficulty to remove IHD stone by ERCP, and the high operative risk. Although IHD stone was not a significant risk factor on our multivariate analysis, it had a tendency to be correlated with stone recurrence. 

The rate of recurrence of symptomatic CBD stone was 10.4% during the follow-up period, consistent with the previous reported rate. However most of the former studies did not distinguish between primary and secondary CBD stones. The shortest period of time for the development of recurrence was 13 months in this study, which was far beyond the minimum required interval of 6 months for the definition of recurrent stone. Interestingly, all the patients developed biliary symptoms with recurrent CBD stone at a follow-up period of over 12 months. 

The potential limitation of the study is substantial dropout of patients due to the retrospective design. However, the subjects were limited to patients with initial CBD stone and subsequent cholecystectomy and who had recurrent symptomatic CBD stone. We used strict criteria based on the hospital data as well as telephone interviews in case of missing data. Another limitation was the possibility of the exclusion of the patients with asymptomatic recurrent CBD stone, which would affect the exact prevalence of recurrent CBD stone, yet the patients with clinically meaningful symptomatic CBD stone were an object of attention in clinical practice. In conclusion, the recurrence of CBD stones in patients who previously underwent cholecystectomy mostly occurred after 12 months. Careful followup might be recommended for the patients with type 1 or 2 periampullary diverticulum and multiple CBD stones. 

## Figures and Tables

**Table 1:** Patient characteristics.

	Recurrence Group (*n* = 15)	Nonrecurrence Group (*n* = 129)	*P* value
Age (years)	66.9 ± 13.5	59 ± 14.5	0.05
Male, % (*n*)	26.7 (4/15)	49.6 (64/129)	0.11
BMI	23.2 ± 2.2	24.5 ± 2.9	0.1
Alcohol, % (*n*)	13.3 (2/15)	19.4 (25/129)	0.74
Smoking, % (*n*)	13.3 (2/15)	20.9 (27/129)	0.74
DM, % (*n*)	20 (3/15)	14.7 (19/129)	0.70
Hypertension, % (*n*)	53.3 (8/15)	32.6 (42/129)	0.15

**Table 2:** Univariate analysis of the risk factors for recurrence of symptomatic CBD stone.

	Recurrence group (*n* = 15)	Nonrecurrence group (*n* = 129)	*P* value
Laboratory data			
AST (IU/L)	248.2 ± 254.0	172.6 ± 236.3	0.25
ALT (IU/L)	168.1 ± 176.4	186.3 ± 205.3	0.74
TB (mg/dL)	2.2 ± 2.1	2.7 ± 2.5	0.49
Amylase (IU/L)	173.0 ± 444.1	170.3 ± 426.4	0.98
Alk-P (IU/L)	566.3 ± 304.2	583.2 ± 496.8	0.90
*γ*-GTP (IU/L)	252.6 ± 222.6	376.3 ± 332.8	0.16
WBC (×10^6^/uL)	10157.3 ± 4332.1	9372.6 ± 4039.2	0.48
Operation type, % (*n*)			
Open	53.3 (8)	31.8 (41)	0.15
Laparoscopic	46.7 (7)	68.2 (88)	
ERCP attempt, % (*n*)			
1	46.7 (7)	75.2 (97)	0.03
≥2	53.3 (8)	24.8 (32)	
CBD stone size (mm)			
<10	20.0 (3)	43.4 (56)	0.10
≥10	80.0 (12)	56.6 (73)	
CBD stone number, % (*n*)			
1	13.3 (2)	47.3 (61)	0.01
≥2	86.7 (13)	52.7 (68)	
Diverticulum, % (*n*)			
Yes	60 (9)	47.3 (61)	0.42
No	40 (6)	52.7 (68)	
Diverticular type, % (*n*)			
Type 1 + Type 2	53.3 (8)	25.6 (33)	0.03
Type 3 + none	46.7 (7)	74.4 (96)	
Intrahepatic stone, % (*n*)			
Yes	20 (3)	4.7 (6)	0.05
No	80 (12)	95.3 (123)	
EST, % (*n*)			
Yes	73.3 (11)	82.2 (106)	0.48
No	26.7 (4)	17.8 (23)	
Lithotripsy, % (*n*)			
Yes	13.3 (2)	6.2 (8)	0.28
No	86.7 (13)	93.8 (121)	
CBD diameter (mm)	20.1 ± 7.1	18.7 ± 12.5	0.67
Distal CBD angle (°)	143.3 ± 15.2	141.5 ± 13.3	0.63
Distal CBD length (mm)	41.5 ± 10.6	38.6 ± 13.6	0.43

AST: aspartate transaminase; ALT: alanine transaminase; TB: total bilirubin; ALK-P: alkaline phosphatase; *γ*-GTP: gamma guanosine triphosphate; WBC: white blood cell count; ERCP: endoscopic retrograde cholangiopancreatography; CBD: common bile duct; EST: endoscopic sphincterotomy.

**Table 3:** Multivariate analysis of the risk factors for recurrence of symptomatic CBD stone.

Variables	Odds ratio (95% CI)	*P* value
Intrahepatic stone	5.6 (0.8–41.7)	0.09
CBD stone number ≥2	6.7 (1.1–28.6)	0.04
Type 1 or 2 diverticulum	73.6 (2.1–2575.3)	0.02
